# Predictors of mortality in patients under treatment for chronic hepatitis B in Ethiopia: a prospective cohort study

**DOI:** 10.1186/s12876-019-0993-1

**Published:** 2019-05-15

**Authors:** Hailemichael Desalegn, Hanna Aberra, Nega Berhe, Girmay Medhin, Bitsatab Mekasha, Svein Gunnar Gundersen, Asgeir Johannessen

**Affiliations:** 1 Medical Department, St. Paul’s Hospital Millennium Medical College, Po. Box 1271, Addis Ababa, Ethiopia; 2 Aklilu Lemma Institute of Pathobiology, Addis Ababa University, Addis Ababa, Ethiopia; 3 Centre for Imported and Tropical Diseases, Oslo University Hospital, Ullevål, Oslo Norway; 4 Research Unit, Sørlandet Hospital HF, Kristiansand, Norway; 5 Department of Global Development and Planning, University of Agder, Kristiansand, Norway; 6 Department of Infectious Diseases, Vestfold Hospital Trust, Tønsberg, Norway

**Keywords:** Hepatitis B, Antiviral therapy, Survival, Resource-limited settings, Epidemiology

## Abstract

**Background:**

Antiviral treatment for chronic hepatitis B (CHB) is largely unavailable in sub-Saharan Africa; hence, little is known about the prognosis after initiating treatment in African CHB patients. In this study we aimed to assess predictors of mortality in one of the largest CHB cohorts in sub-Saharan Africa.

**Methods:**

Two-hundred-and-seventy-six CHB patients who started treatment with tenofovir disoproxil fumarate at a public hospital in Ethiopia between March 18, 2015, and August 1, 2017, were included in this analysis. Patients were followed up until October 1, 2017, and deaths were ascertained through hospital records and telephone interview with relatives. Decompensated cirrhosis was defined as current or past evidence of ascites, either by clinical examination or by ultrasonography. Cox proportional hazard models were used to identify independent predictors of mortality.

**Results:**

Thirty-five patients (12.7%) died during follow-up, 33 of whom had decompensated cirrhosis at recruitment. The median duration from start of treatment to death was 110 days (interquartile range 26–276). The estimated survival was 90.3, 88.2 and 86.3% at 6, 12 and 24 months of follow-up, respectively. Independent predictors of mortality were decompensated cirrhosis (adjusted hazard ratio [AHR] 23.68; 95% CI 3.23–173.48; *p* = 0.002), body mass index < 18.5 kg/m2 (AHR 3.65; 95% CI 1.73–7.72; *p* = 0.001) and older age (per 1-year increment; AHR 1.06; 95% CI 1.02–1.10; *p* = 0.007).

**Conclusions:**

Decompensated cirrhosis, low body mass index and older age were independent predictors of mortality. Improved access to antiviral treatment and earlier initiation of therapy could improve the survival of African CHB patients.

**Trial registration:**

NCT02344498 (ClinicalTrials.gov identifier). Registered 16 January 2015.

## Background

Chronic infection with hepatitis B virus (HBV) is a major cause of mortality worldwide, responsible for 887,000 deaths in 2015. Globally, an estimated 257 million people are living with chronic hepatitis B (CHB), with the highest prevalence rates observed in East Asia and sub-Saharan Africa [1]. In May 2016, the World Health Assembly endorsed the Global Health Sector Strategy on Viral Hepatitis and called for the elimination of viral hepatitis as a public health threat within 2030, by reducing new infections by 90% and mortality by 65% [2]. However, access to hepatitis B testing and treatment is severely restricted in most resource-limited settings, and in 2015 the World Health Organization (WHO) estimated that only 9% of HBV-infected persons had been formally diagnosed, and among those diagnosed only 8% were receiving antiviral treatment [3].

Antiviral therapy of CHB has been shown to effectively prevent complications and improve survival. In a landmark study by Marcellin and colleagues, the development of liver fibrosis and cirrhosis was halted and even reversed in CHB patients who received long-term antiviral therapy [4]. Furthermore, Kim and colleagues have shown that antiviral treatment of CHB prevents the development of hepatocellular carcinoma (HCC) [5]. Unfortunately, in sub-Saharan Africa, treatment is largely unavailable for several reasons, which include lack of funding, shortage of molecular diagnostic tests, regulatory restrictions on antiviral drugs, and lack of commitment from policy makers [6].

Studies of prognostic markers have been the basis for the development of treatment guidelines for CHB from various international liver societies. The Risk Evaluation of Viral Load Elevation and Associated Liver Disease (REVEAL) study, which followed more than 3500 untreated CHB patients in Taiwan for a mean duration of 11 years, demonstrated that higher levels of HBV viral load, elevated alanine aminotransferase (ALT), older age, and male sex were independent predictors of cirrhosis and HCC [7, 8]. Moreover, a follow-up study of 3233 untreated Chinese CHB patients with a mean follow-up of 46.8 months found that male sex, presence of hepatitis symptoms, older age, and low albumin levels were independently associated with shorter survival [9].

More recently, several studies have identified prognostic markers in CHB patients after initiation of antiviral therapy, which can be useful to identify those who require closer follow-up or liver transplantation. Indeed, a recent study from the Republic of Korea found that the presence of ascites and model for end-stage liver disease (MELD) score above 25 increased the risk of mortality and liver transplantation in CHB patients with severe acute exacerbation and hepatic decompensation during antiviral treatment [10].

Studies that assess predictors of mortality in CHB patients in sub-Saharan Africa are absent, and experiences from East Asia and North America are not necessarily applicable in this setting. Better knowledge of the factors that portend a poorer prognosis would allow prioritization of treatment and closer follow-up of individuals at high risk, thus potentially reducing mortality. The present study aimed to assess clinically relevant predictors of mortality in patients who started antiviral treatment in one of the largest treatment programs for CHB in sub-Saharan Africa.

## Methods

### Study setting and participants

A prospective cohort study was established in February 2015 at St. Paul’s Hospital Millennium Medical College, a tertiary care hospital in Addis Ababa, Ethiopia. Ethiopia is the second most populous nation in Africa with a population size close to 100 million [11], and an estimated seroprevalence of hepatitis B surface antigen (HBsAg) of 7.4% [12].

Patients who started antiviral therapy between March 18, 2015, and August 1, 2017, were included in this study. The participants were adults (≥18 years) with chronic hepatitis B, defined as having a positive HBsAg for at least 6 months. Patients were referred to the treatment center based on symptoms of chronic liver disease, or they were asymptomatic individuals found to be HBsAg positive by routine screening at blood banks, antenatal clinics, etc. Patients co-infected with human immunodeficiency virus (HIV) were excluded from the study, as were patients with known HCC or other terminal disease. As this was the first public treatment center in the country, many of the initial patients were enrolled with clinical signs of advanced liver disease. Early experiences from the treatment center was published previously [13].

### Treatment, monitoring and endpoints

Treatment eligibility criteria were based on the European Association for the Study of the Liver (EASL) 2012 guidelines, with some modifications as previously described [13, 14]. The standard therapy was tenofovir disoproxil fumarate (TDF) 300 mg once daily. After initial adherence counselling, TDF was dispensed for 1 month; thereafter the patients received drug supply for 3 months at each follow-up visit.

The following laboratory tests were performed during follow-up:

- Every 3 months: complete blood count, liver enzymes (ALT and aspartate aminotransferase [AST]), creatinine, and HIV rapid test
- Every 6 months: HBsAg, HBV viral load

Liver fibrosis was assessed with transient elastography (Fibroscan 402, Echosense, France) prior to starting treatment and was repeated 6-monthly thereafter. Significant fibrosis (Metavir score F2–4) was defined as > 7.9 kPa and cirrhosis (Metavir score F4) as > 9.9 kPa [15, 16]. Decompensated cirrhosis was defined as current or past evidence of ascites, either by clinical examination or by ultrasonography. Clinical examination was done at the initial visit and was focused on signs of liver disease. Ultrasound of the liver was performed in all patients who started antiviral treatment, and was repeated annually, mainly to detect HCC.

Body mass index (BMI, weight in kilograms divided by height in meters squared) was used to assess nutritional status. Body weight was measured at baseline using a manual scale, and height was measured using a stadiometer mounted on the scale. BMI below 18.5 kg/m2 defined underweight, in accordance with the WHO classification [17].

Deaths were confirmed from hospital records or by telephone interview with the relatives of the patients. Other recorded outcomes included: patients who self-stopped treatment, were transferred to other clinics (HIV or HCC care), or were lost to follow-up. Patients who missed more than two consecutive scheduled visits and could not be traced through phone calls were considered lost to follow-up.

### Statistical analysis

Baseline characteristics were summarized using descriptive statistics. The date of death was recorded from hospital records or by telephone interview with relatives. The date of the last follow-up visit was taken as the censoring date for patients who were transferred out, self-stopped treatment, or were lost to follow-up. Individuals who were alive and in care were right-censored at October 1, 2017.

Kaplan-Meyer survival curves were used to compare mortality across baseline categories of nutritional status and cirrhosis. Multicollinearity was investigated using Spearman’s correlation coefficient with a cutoff at 0.7. Cox proportional hazards models were used to assess factors associated with mortality. Univariable Cox regression analysis was performed for the following baseline variables: gender, age, BMI, ALT, HBV viral load and cirrhosis. Variables with a *P*-value below 0.20 in univariable analysis were included in the multivariable model.

SPSS version 23.0 software (SPSS Inc., Chicago, IL, USA) was used to analyze the data. The level of significance was set at *P* < 0.05. Results were reported in accordance with the Strengthening the Reporting of Observational studies in Epidemiology (STROBE) statement guidelines [18].

### Ethics

The study was approved by the National Research Ethics Review Committee (Ref. No.: 3.10/829/07) in Ethiopia and by the Regional Committees for Medical and Health Research Ethics (Ref. No.: 2014/1146) in Norway. The study was conducted in accordance with the Declaration of Helsinki. Written informed consent was obtained from all study subjects.

## Results

### Patient characteristics

Among 1303 CHB patients enrolled in the program, 276 individuals started antiviral therapy. Of these, 215 (77.9%) were men and 222 (80.4%) were between 18 and 45 years of age. The majority had a normal ALT level (< 40 U/L; *n* = 163, 59.1%) and BMI within the normal range (≥18.5 kg/m2; *n* = 212, 78.8%). Eighty-five patients (30.8%) had a viral load below 2000 IU/ml, and 50 (19.2%) had no or only mild fibrosis based on transient elastography (Table 1).

**Table 1:** Baseline characteristics and associated mortality among patients with chronic hepatitis B who started antiviral treatment in Ethiopia

Characteristics	Number of patients (%) (*n* = 276)	Number of deaths (%) (*n* = 35)
Sex		
Male	215 (77.9)	29 (82.9)
Female	61 (22.1)	6 (17.1)
Age (years)		
18–25	58 (21.0)	4 (11.4)
26–35	101 (36.6)	11 (31.4)
36–45	63 (22.8)	9 (25.7)
> 45	54 (19.6)	11 (31.4)
ALT (U/L)		
< 40	163 (59.1)	19 (54.3)
40–79	78 (28.3)	11 (31.4)
≥ 80	35 (12.7)	5 (14.3)
Body mass index (kg/m2)^a^		
< 18.5	57 (21.2)	14 (41.2)
≥ 18.5	212 (78.8)	20 (58.8)
HBV viral load (IU/m)^b^		
< 2000	85 (30.8)	12 (34.3)
2000-19,999	42 (15.2)	2 (5.7)
≥ 20,000	149 (54.0)	21 (60.0)
Transient elastography (kPa)		
< 8.0	50 (19.2)	1 (3.1)
8.0–9.9	37 (14.2)	1 (3.1)
≥ 10.0	173 (66.5)	30 (93.8)
Cirrhosis assessment		
No cirrhosis	86 (31.2)	1 (2.9)
Compensated cirrhosis^d^	74 (26.8)	1 (2.9)
Decompensated cirrhosis^e^	116 (42.0)	33 (94.3)

^a^n = 269; ^b^n = 251; ^c^n = 260. ^d^Defined as transient elastography ≥10.0 kPa. ^e^Defined as clinical/ultrasonographic evidence of ascites

*ALT* alanine aminotransferase, *HBV* hepatitis B virus

### Mortality

Thirty-five patients (12.7%) died during the follow-up period, 33 of whom had decompensated cirrhosis at recruitment. Four patients (1.4%) were transferred out, 12 (4.3%) were lost to follow-up, and 8 (2.9%) self-stopped treatment. Baseline characteristics of patients who died during follow-up are depicted in Table 1.

The median duration from start of treatment to death was 110 days (interquartile range 26–276). Overall, the estimated survival was 90.3, 88.2 and 86.3% at 6, 12 and 24 months of follow-up, respectively.

### Predictors of mortality

**Fig. 1:**
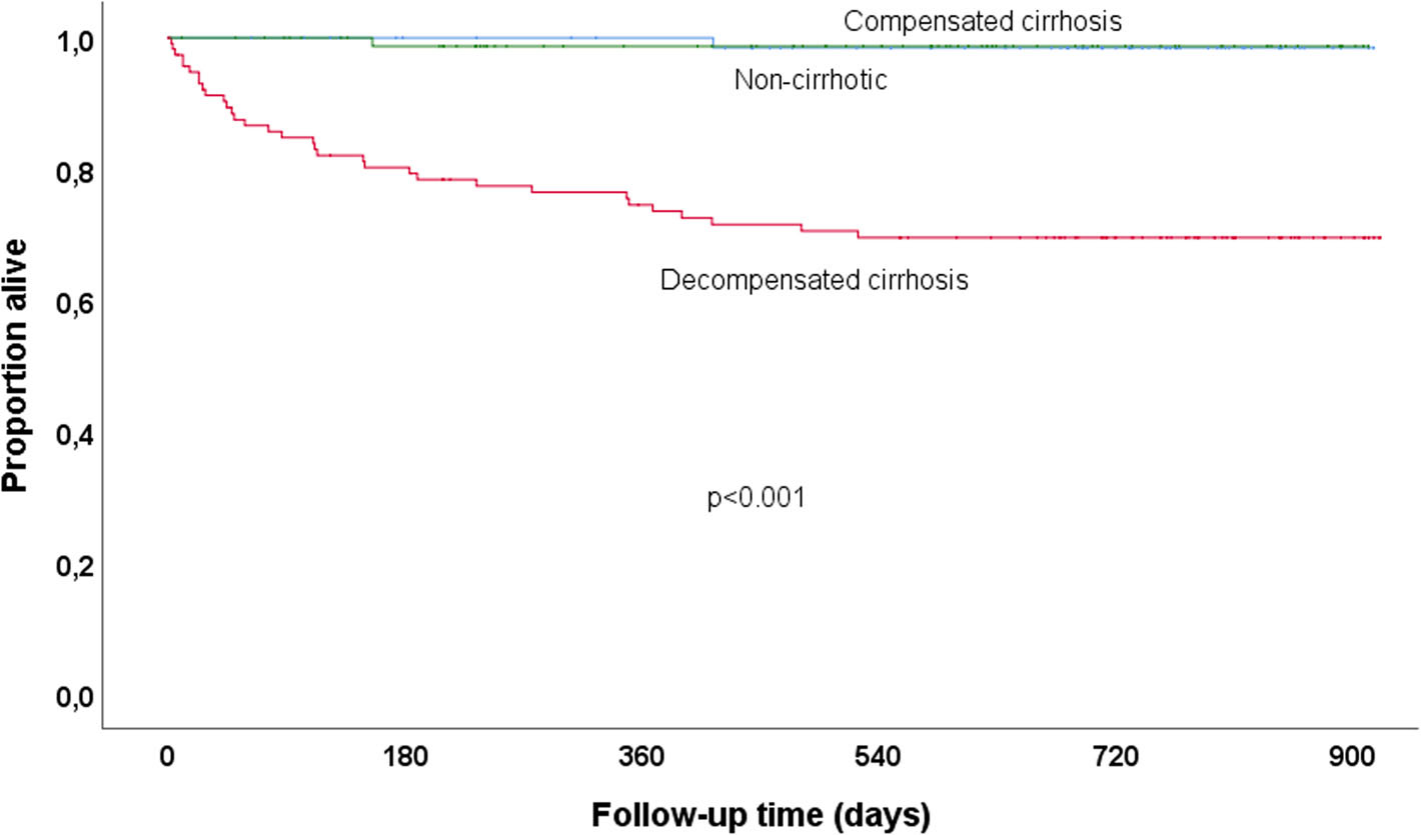
Kaplan-Meier survival curves in patients with decompensated cirrhosis (red), compensated cirrhosis (blue) and no cirrhosis (green) at baseline

Figure 1 illustrates the survival in patients with decompensated cirrhosis compared to those with compensated cirrhosis and no cirrhosis. Estimated survival in patients with decompensated cirrhosis was 75.9, 72.1 and 70.1% at 6, 12 and 24 months of follow-up, respectively. Individuals with compensated cirrhosis, on the contrary, had an excellent prognosis; 100.0, 100.0 and 98.4% were estimated to be alive at 6, 12 and 24 months, respectively.

**Fig. 2:**
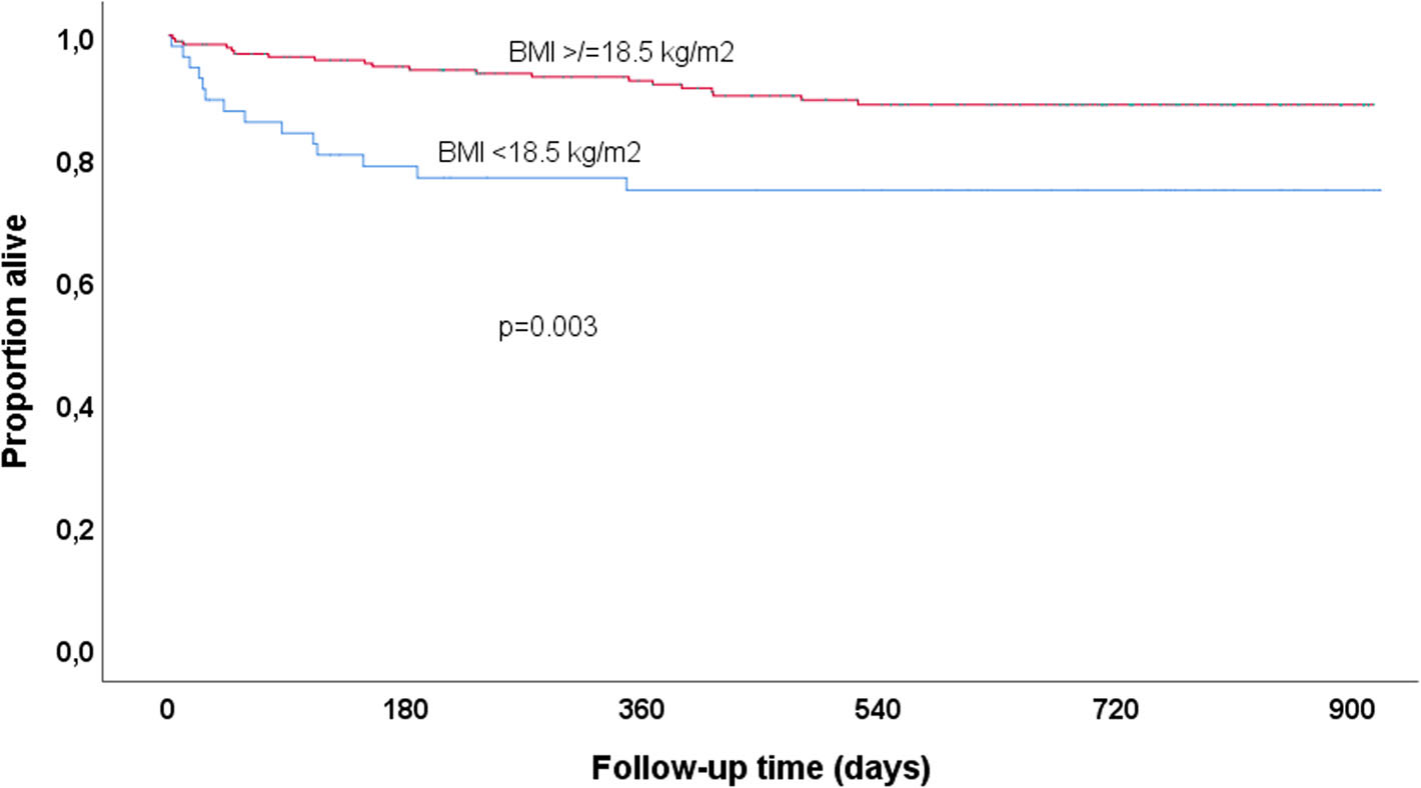
Kaplan-Meier survival curves according to baseline body mass index

Figure 2 compares the survival in patients with BMI below and above 18.5 kg/m2. Estimated survival in underweight patients was 77.0, 75.0 and 75.0% at 6, 12 and 24 months, respectively, compared to 93.6, 91.5 and 89.7% in those with a BMI above 18.5 kg/m2.

Significant predictors of mortality in univariable analysis were low BMI and decompensated cirrhosis. In the adjusted analysis, significant predictors of mortality were decompensated cirrhosis (vs. no cirrhosis; adjusted hazard ratio [AHR] 23.68; 95% CI 3.23–173.48; *p* = 0.002), low body mass index (< 18.5 kg/m2 vs. ≥18.5 kg/m2; AHR 3.65; 95% CI 1.73–7.72; *p* = 0.001), and older age (per 1-year increment; AHR 1.06; 95% CI 1.02–1.10; *p* = 0.007) (Table 2). Transient elastography was not included in the adjusted model as it was strongly correlated with the cirrhosis variable (Spearman’s correlation coefficient 0.808, *p* < 0.001).

**Table 2:** Hazard ratios of mortality according to baseline variables in HBV-infected patients starting antiviral treatment in Ethiopia (*n* = 276)

Baseline variables	Unadjusted	*P*	Adjusted	*P*
	HR (95% CI)		HR (95% CI)	
Sex (male vs. female)	1.44 (0.60–3.48)	0.413	–	
Age (years)	1.03 (1.00–1.06)	0.106	1.06 (1.02–1.10)	0.007
ALT (≥80 U/L vs. < 80 U/L)	1.19 (0.46–3.06)	0.722	–	
BMI (< 18.5 kg/m2 vs. > 18.5 kg/m2)	2.84 (1.43–5.62)	0.003	3.65 (1.73–7.72)	0.001
Transient elastography (kPa)				
< 8.0	1		N/D ^a^	
8.0–9.9	1.29 (0.08–20.57)	0.859		
≥ 10.0	9.06 (1.24–66.43)	0.030		
Cirrhosis assessment				
No cirrhosis	1		1	
Compensated cirrhosis	1.12 (0.07–17.95)	0.935	0.89 (0.06–14.42)	0.937
Decompensated cirrhosis	28.10 (3.84–205.51)	0.001	23.68 (3.23–173.48)	0.002

^a^Transient elastography could not be included in the adjusted model because of a strong correlation with one of the other variables

*HR* hazard ratio, *CI* confidence interval, *ALT* alanine aminotransferase, *BMI* body mass index

## Discussion

The overall mortality in this CHB treatment cohort was 12.7%, with most deaths occurring within the first 6 months after initiation of antiviral treatment. The high early mortality observed in this program was probably a direct consequence of the lack of antiviral therapy in the country. At the time this program was established, antiviral treatment for CHB was unavailable except through the private sector or “black market”, and hence, symptomatic patients desperate for life-saving treatment were overrepresented in the early phase. Indeed, 33 of 35 patients who died had decompensated cirrhosis at enrollment. The same phenomenon was observed in HIV programs a decade ago when patients with advanced, symptomatic disease often were overrepresented in new treatment programs, and the corresponding early mortality was high [19]. Interestingly, in our program the mortality was low after the initial 6 months, even in patients with decompensated cirrhosis, indicating favorable long-term benefit of therapy.

Decompensated cirrhosis was the strongest predictor of mortality in this cohort, increasing the risk of mortality 24-fold. This is in line with a recent study from the Republic of Korea, which showed that the presence of ascites yielded a 10-fold increased risk of death or liver transplantation [10]. Similar studies from low- and middle income countries are scarce, but a small study from India found that a MELD score above 20 was an independent predictor of mortality in CHB patients with decompensated cirrhosis treated with TDF; however, clinical ascites was not evaluated in this study [20].

Low BMI was another strong predictor of mortality in the current study. Although protein-calorie malnutrition is a common feature of advanced chronic liver disease [21], there are limitations with using BMI as a surrogate for nutritional status in this patient group. While it is easy to perform with minimal cost, BMI is inaccurate in patients with ascites and/or edema as the level might be falsely elevated. A recent study from Portugal found that the use of triceps skinfold was a better measure of nutrition in patients with chronic liver disease; this variable also predicted mortality [22]. Unfortunately, this measurement was not obtained in the present study. The association between poor nutritional status and mortality in cirrhotic patients has been well described in studies from Europe, North America and Asia; indeed, malnutrition has been shown to be an independent predictor of various complications including variceal bleeding, refractory ascites and liver related mortality [23–25]. To the best of our knowledge, however, our study is the first to describe the association between malnutrition and mortality in CHB patients in a low-income setting, where malnutrition is common in the general population. Further studies are needed to assess whether nutritional screening, assessment, and intervention might improve survival in this patient population.

Increasing age was also significantly associated with mortality in the present study. This has been consistently reported in previous studies, both in untreated CHB and in treated cohorts, and probably reflects the longer duration of HBV infection [7–9].

The present study identified simple prognostic tools which can be used in settings with limited resources such as Ethiopia. Established scoring tools recommended in international guidelines, such as MELD score or Child-Pugh score, make use of certain laboratory tests such as INR that are not widely available in resource-limited settings. Since ascites and malnutrition can be diagnosed with minimal equipment and costs, early detection of high-risk patients can be ensured even in settings without access to advanced diagnostics. These patients should be prioritized for immediate antiviral treatment and would require closer follow-up in order to manage complications aggressively.

There were some limitations of our study. First, as this was the first and only treatment center in the country, there might be a selection bias towards patients with more advanced disease being recruited. However, this would not have affected the main analysis in the study. Second, mortality might be under-reported in this study, since some patients who were lost to follow-up may have died at home without being reported. Since under-reporting of mortality is likely to be non-differential, the effect estimates in the present study would, if anything, be underestimated [26]. The main strength of our study was that it was carried out in low-middle income country with consistent data collection and included a large volume of patients.

## Conclusion

The current study showed that mortality was high during the initial 6 months after treatment initiation for CHB. Clinical signs of decompensation, older age and low BMI were independent predictors of mortality. Screening CHB patients for the presence of ascites and malnutrition could be a simple, affordable, and practical way to identify high-risk individuals in settings with limited resources. Improved access to diagnostics and earlier initiation of therapy - before progression to decompensated cirrhosis – could improve the survival of chronic hepatitis B patients in sub-Saharan Africa.

## Abbreviations

AHR: Adjusted hazard ratio
ALT: Alanine aminotransferase
AST: Aspartate aminotransferase
BMI: Body mass index
CHB: Chronic hepatitis B
EASL: European association for the study of the liver
HBsAg: Hepatitis B surface antigen
HBV: Hepatitis B virus
HCC: Hepatocellular carcinoma
HIV: Human immunodeficiency virus
MELD: Model for end-stage liver disease
REVEAL: Risk evaluation of viral load elevation and associated liver disease
STROBE: Strengthening the reporting of observational studies in epidemiology
TDF: Tenofovir disoproxil fumarate
WHO: World health organization
